# Genitourethral Reconstruction

**DOI:** 10.1155/2015/535438

**Published:** 2015-02-23

**Authors:** Ralf Herwig, Salvatore Sansalone, Peter Rehder

**Affiliations:** ^1^Department of Reconstructive Urology, Andrology and Men's Health, Vienna Urology Foundation, 1010 Vienna, Austria; ^2^Department of Urology, University of Rome “Tor Vergata”, Via Orazio Raimondo 18, 00173 Rome, Italy; ^3^Department of Urology, Medical University of Innsbruck, 6020 Innsbruck, Austria

To write an editorial is always a good opportunity to say thanks to my coeditors Salvatore Sansalone and Peter Rehder for all the work they have done and to the authors of the many high-class manuscripts.

Reconstructive urology and andrology are still a very new research direction in the field of urology. For this reason we should specially take care to produce innovative research results. These results should not only reflect the so-called mainstream research but should focus on serving the patients.

Here we are particularly keen not to repeat past mistakes from other disciplines.

Reconstructive urology is a highly specialized field of urology that restores both structure and function to the genitourinary tract. Prostate procedures, full or partial hysterectomies, trauma (auto accidents, gunshot wounds, industrial accidents, straddle injuries, etc.), disease, obstructions, blockages (e.g., urethral strictures), and, occasionally, childbirth can require reconstructive surgery. The urinary bladder, ureters (the tubes that lead from the kidneys to the urinary bladder) and genitalia are other examples of reconstructive urology.

The goal in penile reconstruction is to either create or restore both functional and aesthetic phallus. This includes the ability not only to void while standing, from the tip of the phallus, but also to achieve sexual function, with a sensate penis of sufficient bulk to allow for penetration. Generally, the extent of the defect dictates the means of reconstruction we chose for our patients. A surgical defect may range from one involving a single tissue or structure (i.e., skin or urethra) to a total penectomy defect, requiring microsurgical reconstruction. The buried penis presents another interesting problem that demands a somewhat different surgical approach and procedure.

The classical reconstructive surgery in this field is reconstruction after resection of urethral strictures. Here we discuss two different techniques with perineal access in this issue.

Perineal ureterostomy is an option to manage complex and/or recurrent urethral strictures and is necessary after urethrectomy and/or penectomy. Both techniques are associated with about 20% recurrence rate and the patient should be informed about this risk.

Urinary diversion after cystectomy is an important field of reconstruction, which is very often associated with urinary retention and urinary incontinence. In the study of M. Życzkowski et al., the authors present a surgical modification during cystectomy with orthotopic ileal neobladder. The authors could demonstrate that sacrocolpopexy with polypropylene tape as valuable surgical modification during cystectomy with orthotopic ileal bladder is a valuable surgical method which provides patients with a better quality of life.

Another field in this context is transgender surgery. This is a highly complex and specified kind of surgery. For decades, several techniques have been proposed, but, as suggested by C. G. Sutcliffe et al. in a systematic review, no operative standards of care are available in this particular surgical field. Although many procedures are more or less harmonised, several complications are known to occur. Neovaginal prolapse is a relatively rare complication after male-to-female sexual reassignment surgery and tends to be very distressing for both patients and surgeons. In the article of S. Bucci et al. report about the authors incidence in total and partial neovaginal prolapse, how they prevent it, and what their optimal way is to correct it.

Among the hospitalized patients, the admission rate of genitourinary trauma patients is assumed to be 2–10% and one-third of them were found to have an injury on external genitalia. Despite the fact that a classification of trauma is important to establish a strategy of treatment, to date there have been less efforts to make a classification for trauma of external genitalia. To date, there are no specific guidelines for the treatment of severe penile surgery because the injury mechanism is a complex and multifaceted subject. J. H. Kim et al. describe in their review the various penile injuries, which have relatively higher incidence. Physicians should keep in mind that the goal of treatment of penile injury is to achieve normal-like appearance, reduce functional damage such as erectile dysfunction and sensory loss, and minimize the postoperative sequel.

In this light, penile implant surgery often seems the only solution to prevent erectile function in trauma patients.

Nevertheless, nowadays, after 20 years of PDE5 inhibitors, the number of penile implant applications is rising. Although penile implantation remains a final solution for patients with refractory impotence, undesirable postoperative effects, including penile size reduction and cold sensation of the glans penis, remain problematic. Herein G.-L. Hsu et al. found that venous ligations at a retrocoronal level constitute a viable option for reducing the incidence of glanular size reduction. This encouraging preliminary study shows that a combination of venous stripping of the retrocoronal plexus and ligation of the deep dorsal vein and cavernous veins at the penile hilum appears to enhance glanular dimension in implant patients and may treat cold glans syndrome.

Peyronie's disease (PD) is a condition, which is getting more and more common. Nowadays it is assumed that about 10% of all males are suffering from this disease. It is characterized through formation of fibrous plaques which result in penile deformity, pain, and erectile dysfunction. C. Loreto et al. found out that apoptotic cell death occurs in stabilized PD plaques and is partly induced by the intrinsic mitochondrial pathway. The present findings possibly have clinical implications and may help to devise improved treatment strategies and may suggest perhaps new medical treatment options.

Andrology is the medical specialty that deals with male health, particularly relating to the problems of the male reproductive system and urological problems that are unique to men.

It is also known as “the science of men.” It is the counterpart to gynaecology, which deals with medical issues which are specific to the female reproductive system. Andrology has only been studied as a distinct specialty since the late 1960s; the first specialist journal on the subject was the German periodical Andrologie (now called Andrologia), published from 1969 onwards.

All over the world, erectile dysfunction (ED) is considered one of the most diffuse sexual disorders. The prevalence rate of ED increases with age and with concomitant morbidities. Based on these considerations, phosphodiesterase-5 inhibitors (PDE5-i) have become the most popular treatment and are currently the first-line monotherapy for ED. But this category of drugs is not depicted from side effects that could impair pharmacological adherence. In this general context, studies on natural compounds have been conducted with the intention to limit side effects and to maintain efficacy. S. Sansalone et al. demonstrate that patients affected by mild-moderate ED may significantly benefit from oral therapy with this special mixture of natural ingredients by improving sexual and ejaculation function and sexual quality of life. In particular, those patients with moderate arterial dysfunction may significantly benefit from this medication.

A further burden in reduced sperm quality is uni- or bilateral varicocele. Varicoceles are recognized as the most common surgically correctable cause of male infertility, but the exact mechanism of varicocele-induced impairment of spermatogenesis remains a matter of debate. The exact association between reduced male fertility and varicocele is unknown, but a meta-analysis showed that semen improvement is usually observed after surgical correction. The group around Fabrizio Iacono present their experience on patients affected by bilateral varicocele and other scrotal comorbidities treated with surgery with a single scrotal access.

All of these outstanding works are hopefully adapted to expand our knowledge in the field of reconstructive urology and andrology. My coauthors and I are happy about this very successful issue. I wish you all continued enthusiasm and success in your work in this amazing area of medicine.

*Ralf Herwig* *Salvatore Sansalone* *Peter Rehder*

